# Influenza-associated pneumonia hospitalizations in Uganda, 2013-2016

**DOI:** 10.1371/journal.pone.0219012

**Published:** 2019-07-15

**Authors:** Gideon O. Emukule, Barbara Namagambo, Nicholas Owor, Barnabas Bakamutumaho, John T. Kayiwa, Joyce Namulondo, Timothy Byaruhanga, Stefano Tempia, Sandra S. Chaves, Julius J. Lutwama

**Affiliations:** 1 Centers for Disease Control and Prevention, Kenya Country Office, Nairobi, Kenya; 2 Uganda Virus Research Institute, National Influenza Centre (UVRI-NIC), Entebbe, Uganda; 3 Influenza Program, Centers for Disease Control and Prevention, Pretoria, South Africa; 4 Centre for Respiratory Diseases and Meningitis, National Institute for Communicable Diseases of the National Health Laboratory Service, Johannesburg, South Africa; 5 Influenza Division, National Center for Immunization and Respiratory Diseases, US Centers for Disease Control and Prevention, Atlanta, Georgia, United States of America; Public Health England, UNITED KINGDOM

## Abstract

**Background:**

Influenza is an important contributor to acute respiratory illness, including pneumonia, and results in substantial morbidity and mortality globally. Understanding the local burden of influenza-associated severe disease can inform decisions on allocation of resources toward influenza control programs. Currently, there is no national influenza vaccination program in Uganda.

**Methods:**

In this study, we used data on pneumonia hospitalizations that were collected and reported through the Health Management Information System (HMIS) of the Ministry of Health, Uganda, and the laboratory-confirmed influenza positivity data from severe acute respiratory illness (SARI) surveillance in three districts (Wakiso, Mbarara, and Tororo) to estimate the age-specific incidence of influenza-associated pneumonia hospitalizations from January 2013 through December 2016.

**Results:**

The overall estimated mean annual rate of pneumonia hospitalizations in the three districts was 371 (95% confidence interval [CI] 323–434) per 100,000 persons, and was highest among children aged <5 years (1,524 [95% CI 1,286–1,849]) compared to persons aged ≥5 years (123 [95% CI 105–144]) per 100,000 persons. The estimated mean annual rate of influenza-associated pneumonia hospitalization was 34 (95% CI 23–48) per 100,000 persons (116 [95% CI 78–165] and 16 [95% CI 6–28] per 100,000 persons among children aged <5 years and those ≥5 years, respectively). Among children aged <5 years, the rate of hospitalized influenza-associated pneumonia was highest among those who were <2 years old (178 [95% CI 109–265] per 100,000 persons). Over the period of analysis, the estimated mean annual number of hospitalized influenza-associated pneumonia cases in the three districts ranged between 672 and 1,436, of which over 70% represent children aged <5 years.

**Conclusions:**

The burden of influenza-associated pneumonia hospitalizations was substantial in Uganda, and was highest among young children aged <5 years. Influenza vaccination may be considered, especially for very young children.

## Background

Influenza viruses are important contributors to lower respiratory tract infections (LRTI), such as pneumonia, resulting in substantial morbidity and mortality globally every year [1–3]. In general, children aged <2 years, pregnant women, persons with underlying medical conditions, and the elderly are at increased risk of severe disease [4, 5]. In tropical sub-Saharan Africa, other risk factors for influenza severity include human immuno-deficiency virus/acquired immune deficiency syndrome (HIV/AIDS), tuberculosis (TB), malaria, and malnutrition [6–9]. Studies conducted in African countries have estimated rates of influenza-associated hospitalizations that are more than 2-fold higher than estimates from the USA and other industrialized countries [10–14].

In Uganda, the current influenza surveillance system was established in 2006, initially for influenza-like illness (ILI) surveillance. In 2011, severe acute respiratory illness (SARI) surveillance started, partly in response to the global emerging threat of avian and pandemic influenza [15]. As with other tropical and sub-tropical countries [16–18], influenza viruses circulate in Uganda throughout the year with two major peaks and its epidemiology has recently been described [15, 19–21]. However, the burden of influenza-associated pneumonia in Uganda is yet to be reported. Disease burden data are important to inform policy and resources allocation towards influenza control programs as currently there is no national influenza vaccination program in Uganda.

In this study, we used data on pneumonia hospitalizations collected and reported through the Uganda Ministry of Health’s Health Management Information System (HMIS), and the laboratory-confirmed influenza detection data collected from SARI surveillance in three districts (Wakiso, Mbarara, and Tororo) to estimate age-specific incidence of influenza-associated pneumonia hospitalizations from January 2013 through December 2016.

## Methods

### Study sites and population

**Fig 1:**
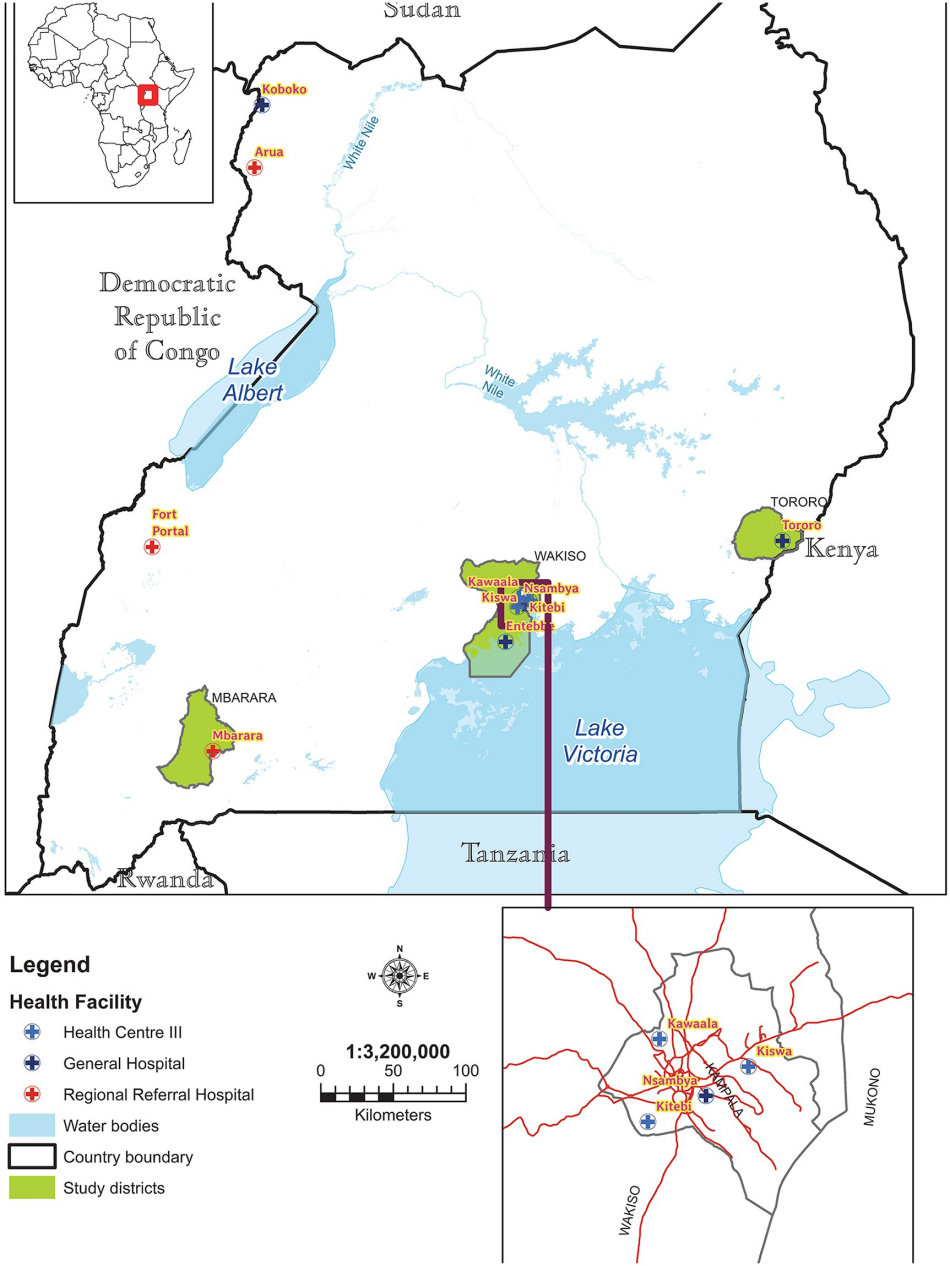
Map of Uganda showing the location of the study sites.

SARI surveillance was established in Entebbe General Hospital (EGH), Mbarara Regional Referral Hospital (MRH), and Tororo General Hospital (TGH) (See Fig 1) in 2011; consistent data were available beginning January 2013. These are the main referral hospitals for residents of Wakiso, Mbarara and Tororo districts respectively. Wakiso is a district in the Central Region of Uganda that partly encircles Kampala, Uganda's capital city. As per the 2014 census, Wakiso district had a population of 1,997,418, with a majority of them (60%) living in urban settlements [22]. Mbarara district is in the Southwestern region of Uganda, and had an estimated population of 472,629 in 2014, of which 60% lived in rural settlements. Tororo district is located in the Eastern part of Uganda and had an estimated population of 517,082 in 2014, of which 86% were living in rural settlements [22]. The total population from these three districts represents 9% of the Ugandan population based on the 2014 census.

### Description of the Uganda Ministry of Health’s Health Management Information System

The Uganda HMIS is an integrated reporting system used by the Ministry of Health (MoH), and other stakeholders to collect information to enable planning, decision making, and monitoring and evaluation of the health care delivery system [23]. The HMIS collects information on a routine basis from every public health unit in all districts within Uganda. Through sets of data collection tools, patient-level data are captured, aggregated into summary reports and submitted first to the district-level unit, and subsequently transmitted to the national-level office. Among the health indicators reported through this system are the service delivery statistics, maternal deaths, and outpatient and inpatient diagnoses (including pneumonia cases as diagnosed by the attending clinician on hospital visit or admission).

Aggregate pneumonia hospitalization data (by sex and age: <5 years, and ≥5 years) are reported from all public and religious-based health facilities on a monthly basis. These monthly summary reports are generated by tallying cases recorded in the hard-copy hospital admission registers at the respective health facilities by the responsible personnel (nurses and health records management staff). These summaries are then entered into an Excel-based template, which is sent by email to the district-level office. Sites that did not have access to computers filled the monthly report summaries on a hard-copy report templates which are then sent to the district-level office for further processing and onward transmission to the national office.

### SARI surveillance and testing for influenza

At each of three hospitals, surveillance officers enrolled patients of all ages who were hospitalized with SARI. The case definition used for SARI enrollment was based on hospitalization for an acute onset of illness (within the last 14 days) with age specific clinical inclusion criteria. For children <5 years old, a modified version of the World Health Organization’s Integrated Management of Childhood Illness (IMCI) definition for pneumonia was used, requiring the presence of cough or difficulty breathing and one or more of the following danger signs: chest in-drawing, stridor, unable to breastfeed or drink, vomits everything, convulsions, lethargy, or unconsciousness [24]. For patients aged ≥5 years, SARI was defined as axillary temperature ≥38°C or history of fever, plus cough or difficulty breathing or shortness of breath.

Patients who were enrolled provided a verbal consent and had nasopharyngeal (NP) and/or oropharyngeal (OP) swabs collected. A structured questionnaire was filled by medical chart abstraction and through direct interview with the patient or caretaker. The NP/OP swabs were combined into a single viral transport media, and sent to the Uganda Virus Research Institute—National Influenza Centre (UVRI-NIC) laboratory in Entebbe where they were tested for influenza A and B by real-time reverse transcription polymerase chain reaction (rtRT-PCR) as previously described [15, 19]. Specimen that tested positive for influenza A were subjected to further testing for subtype characterization at the same laboratory.

### Dealing with gaps, incompleteness, and inconsistency in reporting pneumonia data

**Fig 2:**
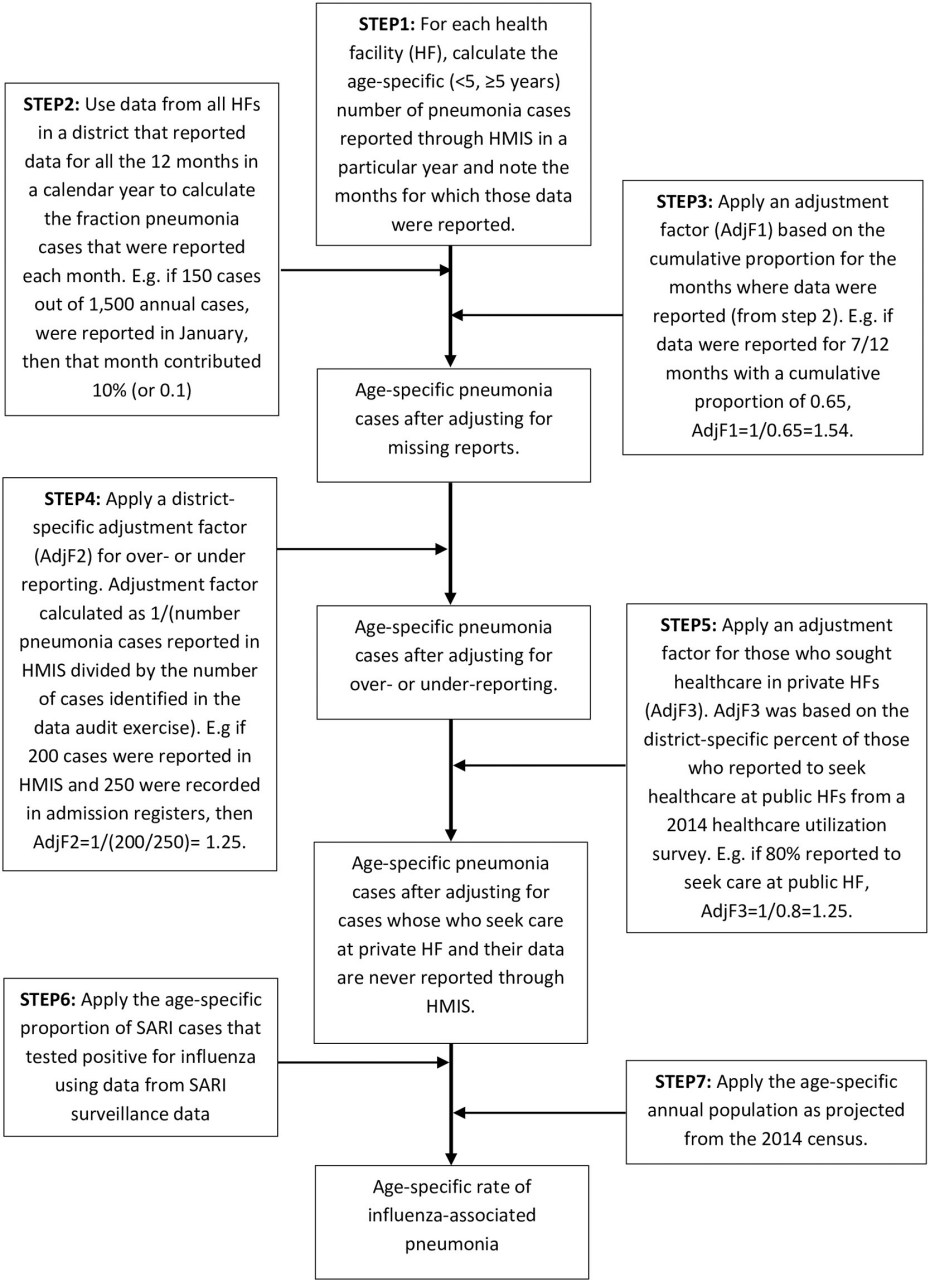
Flow chart showing the steps for estimation.

Three approaches were applied to correct the data on completeness and inconsistencies in reporting as outlined in Fig 2. To adjust for completeness, we adjusted for gaps in each health facility’s monthly reporting using data from facilities, within the same district, with complete data. The district-level monthly distribution of pneumonia was calculated using data from health facilities that had submitted reports for every month in a particular year (100% reporting). For example, consider that data were missing for the months of July, August and September in facility X. If we determined from the district-level data (for the hospitals that had 100% reporting) that the months of July to September contributed 40% of the annual total, the annual total number of pneumonia cases in facility X (adjusted for missing data) will be calculated as sum of cases for the months when data were reported divided by 0.6 (1.0–0.4).

As a second step, to address potential inconsistencies in reporting, a data validation exercise was conducted in a randomly selected number of health facilities. These included the general hospital (where influenza surveillance is ongoing), two other hospitals, and two health centers in each of the three districts. This assessment involved visiting the selected health facilities and conducting a record review of the admission registers to re-tally the number of pneumonia cases that were recorded over a two-year period aggregated by sex and age (<5 years and ≥5 years) as is typically done for HMIS reporting. These re-tallied data were then compared to what was reported to HMIS. An adjustment factor (to correct for potential over/under reporting) was calculated as the reciprocal of the fraction of the total number of pneumonia cases that were reported through HMIS compared to the total number of cases tallied during the data validation.

A third and final step involved adjusting for proportion of pneumonia cases that sought healthcare at private facilities and thus were not necessarily captured through the HMIS (Fig 2). This was done using the proportion of those who reported to seek healthcare (any care seeking) at a public health facility as reported in the healthcare utilization survey that was conducted in 2014 [25]. The adjustment factors used in steps 2 and 3 to estimate the total number of hospitalized pneumonia cases are shown in Table 1.

**Table 1:** Adjustment factors for pneumonia data collected through the Health Management Information System (HMIS) in Uganda, 2013–2016.

Age group	Wakiso District		Mbarara District		Tororo District	
	Adjustment factor for healthcare seeking^a^	Adjustment factor for over/under reporting^b^	Adjustment for healthcare seeking^a^	Adjustment for over/under reporting^b^	Adjustment for healthcare seeking^a^	Adjustment for over/under reporting^b^
<5 years	2.57	1.47	1.97	0.86	1.50	1.11
≥5 years	2.57	0.69	1.97	0.86	1.50	1.07
All ages	2.57	1.08	1.97	0.86	1.50	1.09

^a^Calculated as the reciprocal of the proportion of those who reported to seek healthcare at a government health facility (hospital or health center) based on the healthcare utilization survey conducted in 2014;

^b^Calculated as the reciprocal of the proportion of pneumonia cases that were reported through HMIS relative to the actual number of cases identified in the record count during the validation exercise

### Calculating rates of pneumonia and influenza-associated pneumonia

For each of the three districts selected for this analysis, the estimated number of hospitalized pneumonia cases (incorporating the adjustments outlined above) were calculated. Age-specific (<2, 2–4, 5–14, 15–49, 50–64, and ≥65 years) rates of hospitalized pneumonia were calculated by dividing the number of estimated pneumonia cases by the annual age-specific population [22]. Population estimates for 2013, 2015, and 2016 were projected from 2014 census while assuming an annual population growth rate of 3% [22]. Although pneumonia data reported through HMIS were only available in two broad age categories (<5, and ≥5 years), we assumed that hospitalized patients reported through HMIS had the same age distribution as the SARI cases identified from the influenza surveillance (at the three sites) and applied it to obtain age-specific cases of pneumonia in the finer age categories as defined above. The distribution of SARI cases among patients who were aged ≥13 years were similar to what was published in Kenya for patients who were hospitalized with a respiratory illness [26].

To estimate the age-specific rate of influenza-associated pneumonia hospitalizations, the age-specific (<2, 2–4, and ≥5 years) and annual proportion of SARI cases that tested positive for influenza (Fig A in S2 File) was applied to rate of pneumonia hospitalizations. As influenza testing data were limited, SARI data collected from the three sites (EGH, MRH and TGH) were combined and used to estimate the fraction of SARI cases with influenza. The number of hospitalized influenza-associated pneumonia cases was estimated by applying the estimated age-specific rate of influenza-associated hospitalizations to the age-specific population.

### Data analyses

All data analyses were performed using Stata version 13.0 (StataCorp. 2013. Stata Statistical Software: Release 13. College Station, TX: StataCorp LP). Confidence intervals (CIs) for the rates were estimated by running 1,000 iterations for the respective age categories and each time allowing all of the adjustment factors (for under/over reporting and healthcare utilization), and the proportion of SARI cases that tested positive for influenza to randomly vary within a given range. For each iteration, the parameters were assumed to follow a binomial distribution and centered on the mean. The lower and upper limits of the 95% CI were the 2.5^th^ and 97.5^th^ percentiles of the estimated values obtained from the 1,000 iterations, respectively. All rates were reported per 100,000 persons.

## Ethical considerations

The Ugandan Ministry of Health considered influenza surveillance, and data collected and reported through the HMIS as a routine public health activity and therefore did not require a formal ethical review. SARI data were anonymized upon collection, and authors did not have access to identifying information. Similarly, pneumonia data that were reported through HMIS were anonymized and only available in aggregate form.

## Results

### Descriptive analyses

From 2013 through 2016, a total of 2,662 SARI cases were enrolled at the three SARI surveillance hospitals (EGH = 660, MRH = 675, and TGH = 1,327). Of the SARI cases enrolled, 90% were children aged <5 years, and 201 (7.6%) tested positive for influenza (<2 years = 6.1%; 2–4 years = 10.5%; ≥5 years = 12.0%).

**Fig 3:**
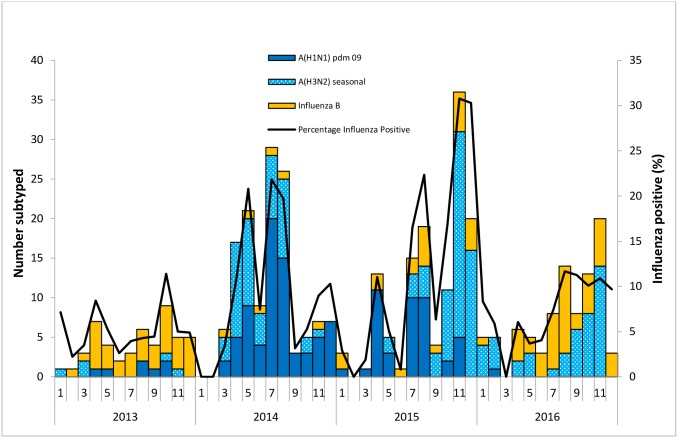
Influenza activity and circulating subtypes in Uganda, 2013–2016.

Of the influenza positive cases identified during this period from these three surveillance sites, 76/201(38%) were influenza A(H3N2), 74/201 (37%) were influenza A(H1N1)pdm09, and 52/201 (26%) were influenza B. A one-year old child was co-infected with influenza A(H3N2) and A(H1N1)pdm09 in 2014. Influenza B was the most commonly circulating type in 2013 (81%), with the influenza A(H1N1)pdm09 dominant in 2014 (63%). Influenza A(H3N2) was the most commonly circulating sub-type in both 2015 (49%) and 2016 (57%) Fig 3 and Table 2.

**Table 2:** Annual distribution of patients who were hospitalized with severe acute respiratory illness (SARI) and tested for influenza at three surveillance hospitals in Uganda, 2013–2016.

Year	2013	2014	2015	2016	2013–2016
	n (%)	n (%)	n (%)	n (%)	n (%)
Patients hospitalized with SARI and tested for influenza					
Entebbe District Hospital	192 (27.5)	224 (26.8)	109 (20.3)	135 (22.9)	660 (24.8)
Mabarara Referral Hospital	138 (19.7)	227 (27.1)	171 (31.8)	139 (23.6)	675 (25.4)
Tororo District Hospital	369 (52.8)	386 (46.1)	257 (47.9)	315 (53.5)	1,327 (49.9)
Total	699 (100.0)	837 (100.0)	537 (100.0)	589 (100.0)	2,662 (100.0)
Patients who tested positive for influenza	37 (5.3)	70 (8.4)	71 (13.2)	23 (3.9)	201 (7.6)
Influenza A	7 (1.0)	67 (8.0)	61 (11.4)	14 (2.4)	149 (5.6)
Influenza B	30 (4.3)	3 (0.4)	10 (1.9)	9 (1.5)	52 (2.0)
Influenza type and sub-type^a^					
Influenza A(H1N1)pndm09	3 (8.1)	44 (62.9)	26 (36.6)	1 (4.4)	74 (36.8)
Influenza A(H3N2)	4 (10.8)	24 (34.3)	35 (49.3)	13 (56.5)	76 (37.8)
Influenza B	30 (81.1)	3 (4.3)	10 (14.1)	9 (39.1)	52 (25.9)

^a^Percentages are based on those who tested positive for influenza type A and/ or B. All the samples that tested positive for influenza A were sub-typed.

### Rates of hospitalized pneumonia

The mean annual rate of hospitalized pneumonia in the three districts was 371 (95% CI 323–434) per 100,000 persons. Rates in children aged <5 years (1,524 [95% CI 1,286–1,849] per 100,000 persons) were higher than in those aged ≥5 years (123 [95% CI 105–144] per 100,000 persons) (Table 3). The estimated mean annual rates of hospitalized pneumonia were highest in Tororo district (647 [95% CI 595–715] per 100, 000 persons) and lowest in Wakiso district (267 [95% CI 223–329] per 100,000 persons) (Table 3 and Table B in S1 File).

**Table 3:** Estimated mean annual age-specific number and rate (per 100,000 persons) of pneumonia hospitalizations in three districts in Uganda, 2013–2016.

District	Age Group	Estimated cases (95% CI)	Rate (95% CI)
Wakiso	<2 years	2958 (2394–3796)	2060 (1667–2643)
	2–4 years	1284 (1039–1647)	596 (482–765)
	<5 years	4241 (3432–5443)	1182 (956–1516)
	5–14 years	850 (665–1045)	139 (109–171)
	15–49 years	199 (156–245)	23 (18–28)
	50–64 years	66 (52–81)	65 (51–80)
	≥65 years	59 (47–73)	101 (79–124)
	≥5 years	1174 (918–1443)	71 (55–87)
	All ages	5415 (4508–6659)	267 (223–329)
Mbarara	<2 years	1157 (991–1330)	3403 (2916–3914)
	2–4 years	504 (432–579)	988 (846–1135)
	<5 years	1660 (1423–1909)	1954 (1674–2247)
	5–14 years	563 (484–642)	389 (334–443)
	15–49 years	133 (114–152)	63 (54–72)
	50–64 years	44 (38–50)	183 (158–209)
	≥65 years	40 (34–45)	284 (245–324)
	≥5 years	779 (669–888)	198 (170–225)
	All ages	2439 (2147–2749)	508 (448–573)
Tororo	<2 years	1590 (1431–1795)	4275 (3848–4826)
	2–4 years	691 (622–779)	1238 (1114–1397)
	<5 years	2280 (2052–2575)	2453 (2208–2770)
	5–14 years	811 (742–909)	511 (468–573)
	15–49 years	190 (174–213)	82 (75–92)
	50–64 years	63 (57–70)	238 (218–266)
	≥65 years	56 (51–63)	367 (335–411)
	≥5 years	1118 (1024–1254)	259 (237–290)
	All ages	3398 (3120–3753)	647 (595–715)
All Combined	<2 years	5704 (4815–6921)	2656 (2242–3222)
	2–4 years	2477 (2091–3004)	769 (649–933)
	<5 years	8181 (6906–9926)	1524 (1286–1849)
	5–14 years	2223 (1890–2594)	243 (207–284)
	15–49 years	522 (443–608)	39 (34–46)
	50–64 years	172 (147–201)	114 (97–133)
	≥65 years	155 (132–180)	176 (150–205)
	≥5 years	3070 (2610–3584)	123 (105–144)
	All ages	11251 (9774–13159)	371 (323–434)

Annual rates of pneumonia hospitalizations varied by year and were highest in 2013 (461 [95% CI 401–538] per 100,000 persons). However, the rates were lowest in the last two years but were comparatively similar (329 [95% CI 287–384] in 2015; and 328 [95% CI 284–384] per 100,000 persons in 2016) (Table B in S1 File).

### Rates of hospitalized influenza-associated pneumonia

For the three districts, the mean annual rate of hospitalized influenza-associated pneumonia was 34 (95% CI 23–45) per 100,000 persons (Table 4). The rates were 7-fold higher in children aged < 5 years (116 [95% CI 78–165] per 100,000 persons) compared to persons aged ≥5 years (16 [95% CI 6–28] per 100,000 persons). Among children aged <5 years, the rates were more than 2-fold higher for children aged <2 years (178 [95% CI 109–265] per 100,000 persons) compared to those who were aged 2–4 years (75 [95% CI 37–125] per 100,000 persons).

**Table 4:** Estimated mean annual age-specific number and rate (per 100,000 persons) of influenza-associated pneumonia hospitalizations in three districts in Uganda, 2013–2016.

District	Age Group	Estimated cases (95% CI)	Rate (95% CI)
Wakiso	<2 years	199 (120–307)	139 (84–214)
	2–4 years	125 (60–216)	58 (28–101)
	<5 years	324 (213–479)	91 (60–134)
	5–14 years	108 (37–193)	18 (7–32)
	15–49 years	26 (9–45)	3 (2–6)
	50–64 years	9 (3–16)	9 (3–16)
	≥65 years	8 (3–14)	14 (6–24)
	≥5 years	151 (52–267)	10 (4–16)
	All ages	474 (321–673)	24 (16–34)
Mbarara	<2 years	76 (48–110)	224 (142–323)
	2–4 years	49 (25–78)	95 (48–153)
	<5 years	125 (85–170)	147 (100–200)
	5–14 years	73 (25–127)	50 (17–88)
	15–49 years	18 (6–30)	9 (3–15)
	50–64 years	6 (2–10)	25 (9–42)
	≥65 years	6 (2–10)	40 (15–67)
	≥5 years	101 (35–176)	26 (9–45)
	All ages	226 (144–317)	47 (30–66)
Tororo	<2 years	107 (67–153)	286 (179–410)
	2–4 years	67 (33–108)	119 (59–193)
	<5 years	173 (117–236)	186 (126–254)
	5–14 years	105 (36–183)	66 (23–116)
	15–49 years	25 (9–43)	11 (4–19)
	50–64 years	9 (3–15)	32 (12–56)
	≥65 years	8 (3–13)	51 (19–86)
	≥5 years	145 (50–254)	34 (12–59)
	All ages	318 (207–447)	61 (40–86)
All Combined	<2 years	382 (234–569)	178 (109–265)
	2–4 years	239 (117–402)	75 (37–125)
	<5 years	620 (414–884)	116 (78–165)
	5–14 years	285 (97–502)	32 (11–55)
	15–49 years	68 (24–118)	6 (2–9)
	50–64 years	24 (8–40)	16 (6–27)
	≥65 years	22 (8–36)	25 (9–41)
	≥5 years	397 (137–697)	16 (6–28)
	All ages	1017 (672–1436)	34 (23–48)

As was the case with pneumonia, the rates of hospitalized influenza-associated pneumonia were highest in Tororo district (61 [95% CI 40–86] per 100,000 persons) and were lowest in Wakiso district (24 [95% CI 16–34] per 100,000 persons). Overall, the annual rates of hospitalized influenza-associated pneumonia were highest in 2015 (41 [95% CI 26–60] per 100,000 persons) and 2014 (35 [95% CI 25–46] per 100,000 persons), but lowest in 2016 (28 [95% CI 18–38], per 100,000 persons) (Table C in S1 File).

### Estimates of hospitalized pneumonia and influenza-associated pneumonia cases

Over the period of analysis, the mean annual number of hospitalized pneumonia cases in the three districts ranged between 9,774 and 13,159 (Table 3). Because of its relatively large population, Wakiso district had the greatest number of cases, ranging from 4,508–6,659 annually. Mbarara and Tororo districts had estimated annual cases of hospitalized pneumonia that ranged between 2,147–2,749, and 3,120–3,753, respectively.

For the three districts, the mean annual number of hospitalized influenza-associated pneumonia cases ranged between 672 and 1,436 (with 414–884 of these among children <5 years). Estimates ranged from 321–673 in Wakiso district, 144–317 in Mbarara, and 207–447 in Tororo district (Table 4).

## Discussion

Using data from three districts studied, we found that influenza virus infections resulted in substantial annual rates of pneumonia hospitalization in Uganda, ranging from 28–41 per 100,000 persons. Rates were particularly high among children <2 years with annual hospitalization rates that ranged from 141–232 per 100,000 children. From a total population of 2,987,129 (who were residents of the three districts), and representing 9% of the overall population of Uganda [22], we estimated that there were 672–1,436 hospitalized influenza-associated pneumonia patients annually. Assuming that these three districts were representative of the general population of Uganda, at a national level—estimated at 34,634,650 in 2014 [22], this would translate to an annual estimate of 7,814–16,698 influenza-associated pneumonia hospitalizations, of which over 70% are children <5 years.

The estimates of hospitalized pneumonia and influenza-associated hospitalized pneumonia that we report here varied by district and by year. Overall, the mean annual rates were the highest in Tororo district (hospitalization rates per 100,000 persons for pneumonia was 595–715, and for influenza-associated pneumonia was 40–86) and lowest in Wakiso district (hospitalization rates per 100,000 persons for pneumonia was 223–329, and for influenza-associated pneumonia was 16–34). Geographical differences in the intensity of influenza viruses circulation and varied care seeking behavior or access to care can explain the difference in rates by area and have been described elsewhere [27]. These differences could also be partly explained by differences in demographic characteristics. Tororo district, for example, has a relatively large rural population compared to Wakiso (86% vs. 40%) and comparatively low health indicators such as high prevalence of malnutrition, which has been shown to be risk factor for pneumonia [6]. The rates of influenza-associated pneumonia hospitalizations were highest in 2015 (41 per 100,000 persons) and lowest in 2013 and 2016. These corresponded to the periods when seasonal influenza A(H3N2) and influenza B were circulating in relatively high proportions compared to the other studied years.

The estimates of influenza-associated pneumonia hospitalizations presented here are comparable to those reported in a global systematic review [28], and analysis of influenza-associated SARI hospitalizations in Africa [11–13, 27]. The rates of influenza-associated pneumonia hospitalizations that we estimated among young children aged <5 years were more than 2-fold higher than rates reported in the US and other industrialized countries [28–32]. On the other hand, our estimates (9–41/100,000 persons) for influenza-associated pneumonia hospitalizations among the elderly (≥65 years) were much lower than rates of influenza-associated hospitalizations reported in the US (186–1100/100,000 person-years) [29, 30]. These low estimates among elderly persons in Uganda could be due to low healthcare seeking behaviors as has previously been suggested in studies that were conducted in other African countries of similar settings [10, 11]. Although direct comparison with other countries’ estimates are challenging due to difference in surveillance case definitions, underlying characteristics of study-population, and methods used, influenza seems to be an important public health problem for Uganda. Further studies using population-based platforms to best characterize the impact of influenza disease burden in Uganda could help policy and stakeholders on resource allocations and interventions.

Our study had some important limitations. Firstly, as mentioned above, we used pneumonia to estimate hospitalizations associated with influenza and may thus have underestimated the true burden of disease. Influenza infections may result in exacerbations of underlying conditions such as asthma, diabetes, and other conditions which are not reflected in these analyses. Secondly, as we used the proportion of influenza positive SARI cases to estimate the fraction of influenza-associated pneumonia hospitalizations, we could have over or underestimated rates as SARI case-definition maybe different from that of pneumonia diagnosed based on clinician’s assessment of patient. Similarly, as pneumonia data were reported only for children <5 years and persons aged ≥5 years, the age distribution of SARI cases was used to define the finer age categories in the pneumonia data. The age-specific estimates of the true burden of influenza-associated pneumonia would vary by how well the SARI cases (identified in the influenza surveillance system) compared to the pneumonia cases (as identified by the healthcare workers at the health facilities). Thirdly, because of limited annual influenza testing by site and the age categories considered, one single proportion for the ages <2, 2–4, and ≥5 years (combining data from the three sites) was calculated and used to estimate the fraction of pneumonia that was associated with influenza. However, the potential effect of this on site-specific estimates may have been limited as exploratory analyses did not show important differences in influenza positivity by site. Lastly, this study did not consider the possible impact of HIV, and other known risk factors such as malnutrition, on pneumonia cases [6, 33]. Further analyses may be warranted not only to assess the extent to which these factors contribute the pneumonia burden in the study district, but also to facilitate extrapolation of burden of disease estimates to the other regions in Uganda.

### Conclusions

Our findings expand knowledge of the impact of influenza infections and highlight a substantial burden of hospitalizations associated with influenza in children aged <5 years in Uganda. These findings suggest that young children, particularly those aged <2 years would benefit most from an annual influenza vaccination program.

## Supporting information

S1 File Supplementary tables. (PDF)

S2 File Supplementary figure. (PDF)
