# Supplementary material for: Influenza-associated pneumonia hospitalizations in Uganda, 2013-2016
Source: PLoS One. 2019 Jul 15;14(7):e0219012. doi: 10.1371/journal.pone.0219012 (PMC6629074; doi:10.1371/journal.pone.0219012)
Supplement: S1 File — (PDF) [file pone.0219012.s001.pdf]

Table A: Annual age-specific population by age group and study site, 2013-2016

Year	Age Group	Wakiso District	Mbarara District	Tororo District	Combined Districts
2013	<2 years	137,239	32,475	35,529	205,243
	2-4 years	205,857	48,711	53,293	307,861
	<5 years	343,096	81,186	88,822	513,104
	5-14 years	585,394	138,516	151,544	875,454
	15-49 years	856,767	202,729	221,797	1,281,293
	50-64 years	96,920	22,934	25,091	144,945
	≥65 years	56,215	13,302	14,553	84,070
	≥5 years	1,595,296	377,481	412,985	2,385,762
	All ages	1,938,392	458,667	501,807	2,898,866
2014	<2 years	141,418	33,463	36,610	211,491
	2-4 years	212,126	50,194	54,915	317,235
	<5 years	353,544	83,657	91,525	528,726
	5-14 years	603,221	142,734	156,159	902,114
	15-49 years	882,859	208,903	228,551	1,320,313
	50-64 years	99,871	23,632	25,855	149,358
	≥65 years	57,926	13,707	14,996	86,629
	≥5 years	1,643,877	388,976	425,561	2,458,414
	All ages	1,997,421	472,633	517,086	2,987,140
2015	<2 years	145,725	34,483	37,725	217,933
	2-4 years	218,587	51,723	56,588	326,898
	<5 years	364,312	86,206	94,313	544,831
	5-14 years	621,592	147,081	160,915	929,588
	15-49 years	909,747	215,266	235,512	1,360,525
	50-64 years	102,913	24,352	26,643	153,908
	≥65 years	59,691	14,125	15,453	89,269
	≥5 years	1,693,943	400,824	438,523	2,533,290
	All ages	2,058,255	487,030	532,836	3,078,121
2016	<2 years	150,163	35,533	38,874	224,570
	2-4 years	225,244	53,298	58,311	336,853
	<5 years	375,407	88,831	97,185	561,423
	5-14 years	640,523	151,561	165,816	957,900
	15-49 years	937,452	221,821	242,684	1,401,957
	50-64 years	106,047	25,094	27,454	158,595
	≥65 years	61,508	14,555	15,924	91,987
	≥5 years	1,745,530	413,031	451,878	2,610,439
	All ages	2,120,937	501,862	549,063	3,171,862
2013-16	<2 years	143,637	33,989	37,185	214,811
	2-4 years	215,454	50,982	55,777	322,213
	<5 years	359,090	84,970	92,962	537,022
	5-14 years	612,683	144,973	158,609	916,265
	15-49 years	896,707	212,180	232,136	1,341,023
	50-64 years	101,438	24,003	26,261	151,702
	≥65 years	58,835	13,923	15,232	87,990
	≥5 years	1,669,662	395,078	432,237	2,496,977
	All ages	2,028,752	480,048	525,198	3,033,998

Table B: Annual age-specific rate (per 100,000 persons) of pneumonia hospitalizations by age group and study site, 2013-2016

Year	Age Group	Wakiso District	Mbarara District	Tororo District	Combined Districts
		Rate (95% CI)	Rate (95% CI)	Rate (95% CI)	Rate (95% CI)
2013	<2 years	2561 (2084 - 3307)	4610 (3982 - 5311)	5593 (5044 - 6273)	3410 (2897 - 4137)
	2-4 years	741 (603 - 957)	1337 (1154 - 1539)	1618 (1458 - 1814)	987 (838 - 1197)
	<5 years	1469 (1195 - 1897)	2646 (2285 - 3048)	3208 (2893 - 3598)	1956 (1662 - 2374)
	5-14 years	135 (106 - 165)	444 (385 - 506)	660 (604 - 737)	274 (237 - 318)
	15-49 years	22 (17 - 27)	72 (62 - 82)	106 (97 - 119)	44 (38 - 51)
	50-64 years	62 (50 - 77)	210 (184 - 240)	303 (279 - 339)	127 (111 - 148)
	≥65 years	98 (79 - 121)	324 (279 - 369)	468 (427 - 523)	198 (171 - 230)
	≥5 years	68 (54 - 84)	226 (196 - 257)	334 (306 - 373)	139 (120 - 161)
	All ages	316 (262 - 392)	654 (579 - 737)	843 (774 - 923)	461 (401 - 538)
2014	<2 years	1942 (1557 - 2491)	4217 (3578 - 4880)	4040 (3622 - 4588)	2665 (2234 - 3232)
	2-4 years	562 (451 - 720)	1224 (1038 - 1415)	1171 (1049 - 1329)	772 (647 - 936)
	<5 years	1114 (893 - 1429)	2421 (2054 - 2800)	2319 (2079 - 2633)	1530 (1282 - 1855)
	5-14 years	145 (114 - 179)	376 (320 - 431)	535 (492 - 601)	249 (212 - 292)
	15-49 years	24 (19 - 29)	61 (52 - 69)	86 (79 - 97)	40 (34 - 47)
	50-64 years	69 (54 - 84)	174 (149 - 199)	244 (225 - 275)	116 (98 - 135)
	≥65 years	104 (82 - 128)	278 (234 - 314)	381 (347 - 427)	179 (152 - 209)
	≥5 years	74 (58 - 91)	191 (162 - 219)	271 (249 - 304)	127 (108 - 148)
	All ages	258 (214 - 317)	586 (510 - 662)	633 (583 - 701)	375 (324 - 438)
2015	<2 years	1879 (1521 - 2401)	2454 (2100 - 2795)	3712 (3356 - 4227)	2287 (1930 - 2779)
	2-4 years	544 (440 - 695)	712 (610 - 811)	1077 (974 - 1226)	663 (559 - 805)
	<5 years	1078 (873 - 1377)	1409 (1206 - 1604)	2131 (1927 - 2427)	1313 (1108 - 1595)
	5-14 years	133 (103 - 163)	378 (322 - 428)	470 (430 - 528)	230 (195 - 268)
	15-49 years	22 (17 - 27)	61 (53 - 70)	75 (69 - 85)	37 (32 - 43)
	50-64 years	63 (49 - 77)	177 (152 - 202)	222 (203 - 248)	108 (92 - 127)
	≥65 years	96 (74 - 118)	277 (241 - 319)	337 (311 - 382)	166 (142 - 195)
	≥5 years	68 (53 - 83)	192 (164 - 218)	238 (218 - 268)	117 (99 - 136)
	All ages	247 (208 - 301)	407 (359 - 456)	573 (528 - 637)	329 (287 - 384)
2016	<2 years	1887 (1530 - 2414)	2455 (2111 - 2819)	3839 (3442 - 4316)	2315 (1953 - 2807)
	2-4 years	546 (443 - 698)	713 (614 - 818)	1112 (997 - 1248)	671 (566 - 812)
	<5 years	1083 (878 - 1384)	1410 (1213 - 1618)	2202 (1975 - 2475)	1328 (1121 - 1611)
	5-14 years	144 (112 - 176)	361 (311 - 410)	393 (359 - 440)	221 (186 - 259)
	15-49 years	23 (18 - 28)	59 (51 - 67)	63 (58 - 71)	36 (30 - 42)
	50-64 years	67 (52 - 83)	172 (148 - 196)	186 (168 - 208)	105 (88 - 122)
	≥65 years	105 (82 - 128)	262 (227 - 296)	289 (264 - 321)	161 (136 - 187)
	≥5 years	73 (57 - 89)	184 (158 - 209)	200 (182 - 223)	112 (95 - 132)
	All ages	252 (210 - 308)	401 (355 - 452)	554 (506 - 612)	328 (284 - 384)
2013-16	<2 years	2060 (1667 - 2643)	3403 (2916 - 3914)	4275 (3848 - 4826)	2656 (2242 - 3222)
	2-4 years	596 (482 - 765)	988 (846 - 1135)	1238 (1114 - 1397)	769 (649 - 933)
	<5 years	1182 (956 - 1516)	1954 (1674 - 2247)	2453 (2208 - 2770)	1524 (1286 - 1849)
	5-14 years	139 (109 - 171)	389 (334 - 443)	511 (468 - 573)	243 (207 - 284)
	15-49 years	23 (18 - 28)	63 (54 - 72)	82 (75 - 92)	39 (34 - 46)
	50-64 years	65 (51 - 80)	183 (158 - 209)	238 (218 - 266)	114 (97 - 133)
	≥65 years	101 (79 - 124)	284 (245 - 324)	367 (335 - 411)	176 (150 - 205)
	≥5 years	71 (55 - 87)	198 (170 - 225)	259 (237 - 290)	123 (105 - 144)
	All ages	267 (223 - 329)	508 (448 - 573)	647 (595 - 715)	371 (323 - 434)

Table C: Annual age-specific rate (per 100,000 persons) of influenza-associated pneumonia hospitalizations by age group and study site, 2013-2016

Year	Age Group	Wakiso District	Mbarara District	Tororo District	Combined Districts
		Rate (95% CI)	Rate (95% CI)	Rate (95% CI)	Rate (95% CI)
2013	<2 years	116 (59 - 192)	207 (118 - 327)	251 (141 - 380)	153 (82 - 246)
	2-4 years	41 (15 - 74)	74 (27 - 130)	89 (34 - 160)	54 (20 - 98)
	<5 years	71 (42 - 110)	127 (79 - 182)	154 (94 - 222)	94 (57 - 141)
	5-14 years	19 (9 - 32)	62 (27 - 101)	92 (39 - 149)	39 (17 - 64)
	15-49 years	4 (2 - 6)	10 (5 - 17)	15 (7 - 24)	7 (3 - 11)
	50-64 years	10 (5 - 16)	31 (14 - 48)	44 (20 - 72)	19 (9 - 31)
	≥65 years	15 (8 - 24)	46 (23 - 76)	69 (28 - 110)	29 (14 - 47)
	≥5 years	10 (5 - 17)	32 (14 - 52)	47 (20 - 76)	20 (9 - 33)
	All ages	21 (14 - 30)	49 (31 - 67)	66 (43 - 92)	33 (22 - 47)
2014	<2 years	135 (88 - 192)	293 (204 - 392)	282 (197 - 380)	185 (125 - 256)
	2-4 years	65 (40 - 100)	142 (88 - 204)	135 (82 - 192)	89 (55 - 132)
	<5 years	93 (65 - 129)	203 (149 - 260)	194 (147 - 245)	128 (92 - 170)
	5-14 years	17 (7 - 29)	43 (17 - 75)	60 (24 - 102)	28 (11 - 49)
	15-49 years	3 (2 - 5)	7 (3 - 12)	10 (4 - 17)	5 (2 - 8)
	50-64 years	9 (4 - 14)	22 (9 - 38)	28 (12 - 50)	14 (6 - 23)
	≥65 years	13 (6 - 21)	37 (15 - 59)	47 (21 - 74)	22 (10 - 36)
	≥5 years	9 (4 - 15)	22 (9 - 38)	31 (12 - 52)	15 (6 - 25)
	All ages	24 (17 - 32)	54 (38 - 71)	60 (42 - 79)	35 (25 - 46)
2015	<2 years	190 (129 - 278)	250 (169 - 337)	377 (258 - 511)	232 (157 - 328)
	2-4 years	78 (48 - 121)	103 (62 - 149)	154 (94 - 227)	95 (58 - 144)
	<5 years	123 (92 - 172)	162 (121 - 205)	243 (180 - 319)	150 (112 - 203)
	5-14 years	19 (0 - 41)	55 (0 - 115)	68 (0 - 148)	33 (0 - 71)
	15-49 years	4 (0 - 7)	9 (0 - 19)	12 (0 - 24)	6 (0 - 12)
	50-64 years	10 (0 - 20)	29 (0 - 54)	34 (0 - 72)	17 (0 - 34)
	≥65 years	16 (0 - 31)	43 (0 - 85)	52 (0 - 111)	26 (0 - 53)
	≥5 years	10 (0 - 21)	28 (0 - 59)	35 (0 - 76)	17 (0 - 36)
	All ages	30 (21 - 43)	52 (28 - 79)	72 (44 - 110)	41 (26 - 60)
2016	<2 years	115 (59 - 193)	150 (79 - 240)	235 (119 - 368)	141 (73 - 231)
	2-4 years	48 (11 - 105)	62 (16 - 132)	98 (25 - 191)	59 (14 - 125)
	<5 years	75 (39 - 122)	97 (53 - 156)	153 (84 - 228)	92 (49 - 146)
	5-14 years	17 (10 - 26)	43 (25 - 62)	47 (29 - 66)	26 (16 - 38)
	15-49 years	3 (2 - 5)	8 (5 - 10)	8 (5 - 11)	5 (3 - 7)
	50-64 years	9 (5 - 13)	20 (12 - 32)	22 (15 - 33)	13 (8 - 19)
	≥65 years	14 (9 - 20)	35 (21 - 49)	38 (26 - 51)	21 (14 - 30)
	≥5 years	9 (5 - 13)	22 (13 - 32)	24 (15 - 34)	14 (8 - 20)
	All ages	21 (13 - 29)	36 (25 - 49)	47 (31 - 62)	28 (18 - 38)
2013-16	<2 years	139 (84 - 214)	224 (142 - 323)	286 (179 - 410)	178 (109 - 265)
	2-4 years	58 (28 - 101)	95 (48 - 153)	119 (59 - 193)	75 (37 - 125)
	<5 years	91 (60 - 134)	147 (100 - 200)	186 (126 - 254)	116 (78 - 165)
	5-14 years	18 (7 - 32)	50 (17 - 88)	66 (23 - 116)	32 (11 - 55)
	15-49 years	3 (2 - 6)	9 (3 - 15)	11 (4 - 19)	6 (2 - 9)
	50-64 years	9 (3 - 16)	25 (9 - 42)	32 (12 - 56)	16 (6 - 27)
	≥65 years	14 (6 - 24)	40 (15 - 67)	51 (19 - 86)	25 (9 - 41)
	≥5 years	10 (4 - 16)	26 (9 - 45)	34 (12 - 59)	16 (6 - 28)
	All ages	24 (16 - 34)	47 (30 - 66)	61 (40 - 86)	34 (23 - 48)
