# Supplementary figures and images for: Influenza-associated pneumonia hospitalizations in Uganda, 2013-2016
Source: PLoS One. 2019 Jul 15;14(7):e0219012. doi: 10.1371/journal.pone.0219012 (PMC6629074; doi:10.1371/journal.pone.0219012)

**Fig A:** Annual age-specific percentage of SARI patients who tested positive for influenza, 2013-2016

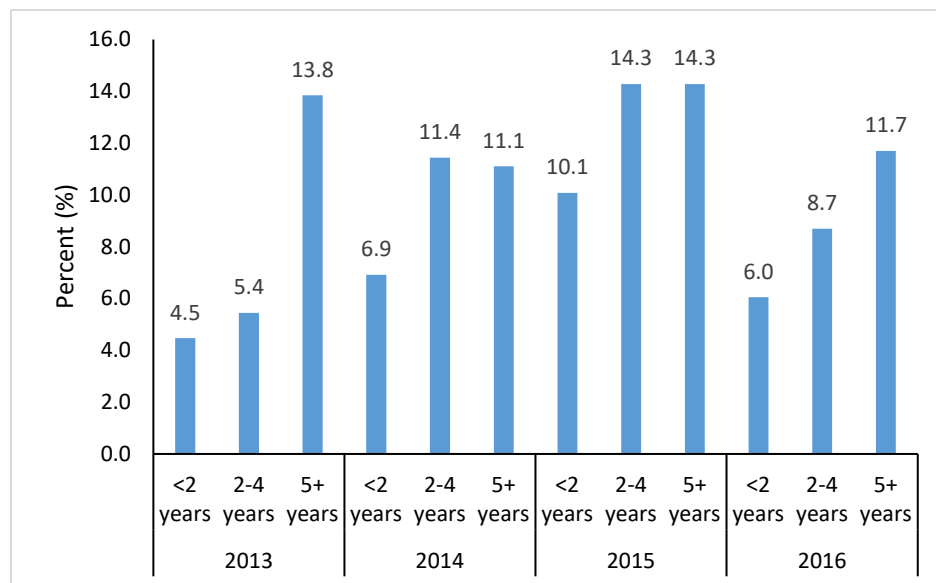

Supplement: S2 File — (PDF) [file pone.0219012.s002.pdf]
